# Emerging Role of Dermal White Adipose Tissue in Modulating Hair Follicle Development During Aging

**DOI:** 10.3389/fcell.2021.728188

**Published:** 2021-10-15

**Authors:** Jian Chen, Zhe-Xiang Fan, De-Cong Zhu, Yi-Long Guo, Ke Ye, Damao Dai, Zhi Guo, Zhi-Qi Hu, Yong Miao, Qian Qu

**Affiliations:** Department of Plastic and Aesthetic Surgery, Nanfang Hospital of Southern Medical University, Guangzhou, China

**Keywords:** aging, hair follicle, dermal white adipose tissue, inflamm-aging, microenvironment

## Abstract

Hair follicle stem cells are extensively reprogrammed by the aging process, manifesting as diminished self-renewal and delayed responsiveness to activating cues, orchestrated by both intrinsic microenvironmental and extrinsic macroenvironmental regulators. Dermal white adipose tissue (dWAT) is one of the peripheral tissues directly adjacent to hair follicles (HFs) and acts as a critical macroenvironmental niche of HF. dWAT directly contributes to HF aging by paracrine signal secretion. However, the altered interrelationship between dWAT and HF with aging has not been thoroughly understood. Here, through microdissection, we separated dWAT from the skin of aged mice (18 months) and young mice (2 months) in telogen and depilation-induced anagen for transcriptome comparing. Notably, compared with young dWAT, aberrant inflammatory regulators were recapitulated in aging dWAT in telogen, including substantial overexpressed inflammatory cytokines, matrix metalloproteinases, and prostaglandin members. Nonetheless, with anagen initiation, inflammation programs were mostly abolished in aging dWAT, and instead of which, impaired collagen biosynthesis, angiogenesis, and melanin synthesis were identified. Furthermore, we confirmed the inhibitory effect on hair growth of CXCL1, one of the most significantly upregulated inflammation cytokines in aging dWAT. Besides this, we also identified the under-expressed genes related to Wnt signaling fibroblast growth factor family members and increased BMP signaling in aging dWAT, further unraveling the emerging role of dWAT in aging HFs malfunction. Finally, we proved that relieving inflammation of aging dWAT by injecting high-level veratric acid stimulated HF regenerative behavior in aged mice. Concomitantly, significantly decreased TNF-a, CCL2, IL-5, CSF2, and increased IL10 in dWAT was identified. Overall, the results elaborated on the complex physiological cycling changes of dWAT during aging, providing a basis for the potential regulatory effect of dWAT on aging HFs.

## Introduction

**FIGURE 1:**
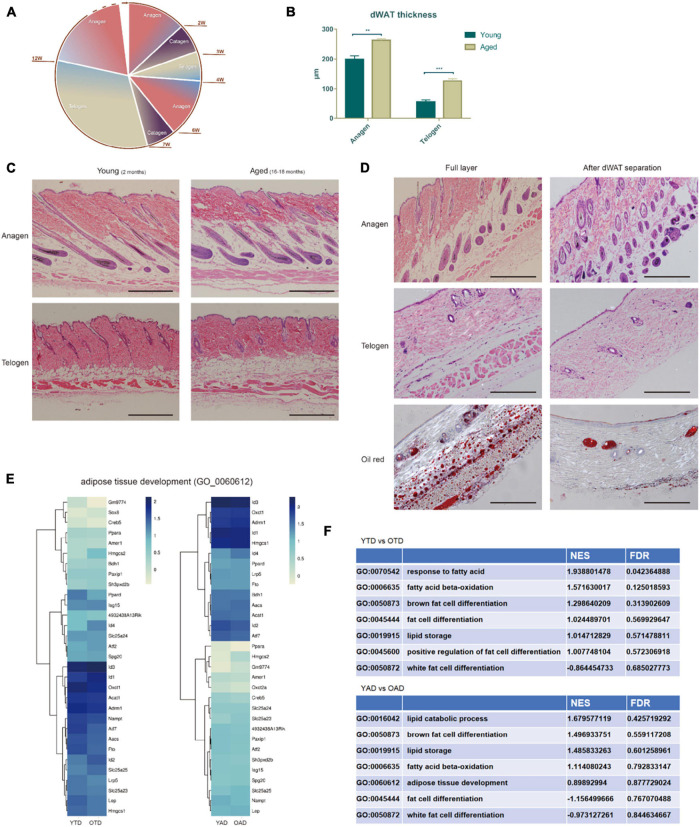
Aging dWAT undergoes structural remodeling with HF defective growth. **(A)** Decelerated hair cycling with aging. **(B,C)** dWAT thickness significantly increased with aging. Comparing to young mice, the dermis thickness decreased while the dWAT thickness increased at both anagen and telogen in aged mice. **P < 0.01, ***P < 0.001. *n* = 10 **(D)** By microdissection, the subcutaneous adipose tissue and panniculus carnosus were in turn removed, and dWAT was then harvested by slightly scraping from the bottom of HFs. **(E)** Unsupervised clustering of OAD/YAD, OTD/YTD samples based on GO signature—adipose tissue development. No significant difference was indicated between each comparison. Dark blue represents high expression level and yellow represents low expression level. **(F)** GSEA analysis of adipose-related terms all exhibited no significant enrichment. All terms’ FDR exceeds 0.05. NES, Normalized Enrichment score. FDR, False discovery rate.

Progressive deterioration in the regenerative potential of stem cells is a hallmark of aging, which results in the failure to maintain proper tissue homeostasis. Hair follicles (HFs) are independent autonomous stem cell niches and undergo continuous regenerative cycling during their lifespan. With aging, HF have diminished self-renewing capacity, manifesting as cycling defects and poor responsiveness to activating stimuli. HF cycling slows down (Figure 1A) with aging and gradually turns into senescent alopecia. Proper homeostasis between inhibiting signals and activating signals underlies continued HF growth. With aging, inhibitory signals transcend the activatory signals, becoming the dominant environmental factor for HFs (Lei and Chuong, 2016).

The aberrant repressive signalings come from both the intrinsic niche microenvironment and extrinsic macroenvironment. In intrinsic factors, transcription nuclear factor of activated T cells, cytoplasmic 1 (Nfatc1) is a downstream target of BMP signaling, and its upregulation in aging HFs considerably contribute to the HF aging process (Keyes et al., 2013). Besides, fork-head box C1 (Foxc1) (Lay et al., 2016; Wang L. et al., 2016), LIM homeobox 2 (Lhx2) (Folgueras et al., 2013), collagen type XVII alpha 1 (Col17a1) (Matsumura et al., 2016) are all crucial to aging properties of HFs. Regarding macroenvironmental factors, multiple cues from varied tissues are integrated to govern HF maintenance. Surrounding adipose tissues, blood vessels (Mecklenburg et al., 2000; Yano et al., 2001; Xiao et al., 2013), nerves (Brownell et al., 2011; Zhang et al., 2020), adjacent cells [epidermal cells (Doles et al., 2012) and immune cells (Chen et al., 2015; Wang et al., 2019, 2017)] are all involved in the extra-follicular macroenvironment regulators. Among these regulators, dermal white adipose tissue (dWAT) acts as a significant niche supporting HF development and its malfunction is directly responsible for HF aging (Guerrero-Juarez and Plikus, 2018).

Traditionally, adipose tissue performs basic nutritional and metabolic functions in the body. But in the skin, dWAT also plays an important tissue-specific role: it provides essential factors modulating periodic HF growth, responds to skin bacterial infection, and promotes wound healing (Zwick et al., 2018). The tight relationship between dWAT and HF has been established by various mouse models with defects in dWAT. Mice lacking Early B cell factor 1 (Ebf1-/-) lost adipocyte precursors in their skin, resulting in telogen retention and failure in anagen re-entry (Festa et al., 2011). Diacylglycerol acyltransferase (DGAT) 1/2 deficient mice indicated impaired triglyceride metabolism and reduced dWAT, resulting in a destroyed skin barrier and hair loss (Chen et al., 2002; Stone et al., 2004). Peroxisome proliferator-activated receptor-gamma (PPARγ) serves as a dominant regulator of adipocytes, and its ablation leads to total lipoatrophy and temporary postnatal delayed HF morphogenesis (Sardella et al., 2017). In terms of mechanism, it has been proved that during late catagen/early telogen, high levels of BMP2 derived from mature adipocytes in dWAT maintains HF in a quiescent, refractory telogen state (Plikus et al., 2008). Nonetheless, in late competent telogen, adipocyte progenitors produce platelet-derived growth factor subunit-α (PDGFa), directly stimulating a new HF cycle (Plikus et al., 2008).

More importantly, in the aging process of HF, dWAT was also proved to exert noticeable functions. Aging dWAT overexpressed inhibiting signals Bmp2, Dkk1, and Sfrp4 during early anagen but decreased activating signal follistatin during late telogen and early anagen, causing repressive HF cycling behavior in aging mice. After transplanting aging skin to young donor mice, defective HF growth in aging mice was partially recovered under the modulation of young dWAT (Chen et al., 2014). dWAT has a well-established role in HF aging but the prise interplay between it and HF in aging phenotype remained largely unclear. Such being the case, to probe more deeply into the role changes of dWAT during aging, we separated dWAT in telogen/anagen of elderly/young mice for transcriptome analysis and further exploration.

## Results

### Hair Growth Wanes as Aging Progresses With Dermal White Adipose Tissue Rewiring

Most HFs in old mice are in refractory telogen, which hardly initiates proliferative machinery into action. Moreover, in contrast to synchronous anagen activation in young mice, aged HF wave propagation becomes increasingly asynchronous and fragmented. To interrogate HF cycling-dependent variations between aged (16–18 months) and young (2 months) mice, we induced a new hair growth cycle *via* depilation (hair plucking using a mixture of wax and rosin). As a result, young mice entered anagen within 8–10 days post-depilation, whereas old mice took 12–13 days for anagen entry, characterized by more sparse hairs than in young mice. Upon depilation stimuli, aged HFs exhibit activation more synchronously, mimicking the response fashion of young mice, though over a longer period. This result indicated that the responsiveness of HFs to tissue-regenerating cues were still retained with aging and could be activated by exogenous stimulus.

Dermal white adipose tissue regenerates in parallel with the hair cycle, which preceed adipogenesis in anagen and lipolysis in telogen (Zwick et al., 2018). First, we compared the thickness of dWAT of old and young mice at anagen/telogen. Intriguingly, we found that aging dWAT thickness increased at both anagen and telogen. Especially at telogen, the thickness of aged dWAT is much higher than that of young dWAT (Figures 1B,C). The results were different from the traditional view that dWAT thickness decreases with aging. After repeating a many slices we confirmed that the actual thinning tissue in the aging skin was the dermis rather than the dWAT, and the thinner dWAT may be caused by the cutting angle of the HE slice. Coincidentally, we found that Salzer et al. also identified increased thickness of dWAT and thinner dermis in aging mice in 2018 (Salzer et al., 2018). Using Single-cell transcriptomics, they proved that skin fibroblasts acquired an adipogenic profile with aging and transformed into adipogenic lineage cells, resulting in an increase in the number of adipogenic lineage cells and thickened dWAT (Salzer et al., 2018).

Aiming to further explore cycling behavior differences of dWAT between aged and young mice, we processed a transcriptome analysis of dWAT in the telogen/anagen phase of old and young mice. Old mice (18 months) and young mice (2 months) at telogen were selected and were respectively used for new anagen induction *via* hair plucking. Since dWAT is a mix of various types of cells (adipocyte lineage, immune cells, vascular cells, and so on), we chose microdissection to separate complete dWAT from mouse skin. After carefully removing the subcutaneous adipose tissue and panniculus carnosus in turn, dWAT were harvested by slightly scraping from the bottom of HFs (Figure 1D). Therefore, four groups were enrolled in this study: OTD (old mice, telogen, dWAT), YTD (young mice, telogen, dWAT), OAD (old mice, anagen, dWAT), YAD (young mice, anagen, dWAT).

Concerning the increased dWAT thickness of aged mice, we speculated whether there was excessive adipogenesis in aging dWAT. Therefore, we then conducted gene ontology (GO) and gene set enrichment analysis (GSEA) of these data. However, based on the results, we found no significant difference in adipose tissue development between aging and young dWAT at both anagen and telogen (Figures 1E,F and Supplementary Figure 1). This result is in line with the most recent study by Salzer showing that the thickness of dWAT significantly increasing with aging (Salzer et al., 2018) is mainly due to the replenishment of other transformed adipogenic lineage cells (aging dermal fibroblasts) rather than enhanced development of the original adipose tissue.

### Screening Aging-Related Dermal White Adipose Tissue Changes With Hair Follicles Cycling

**FIGURE 2:**
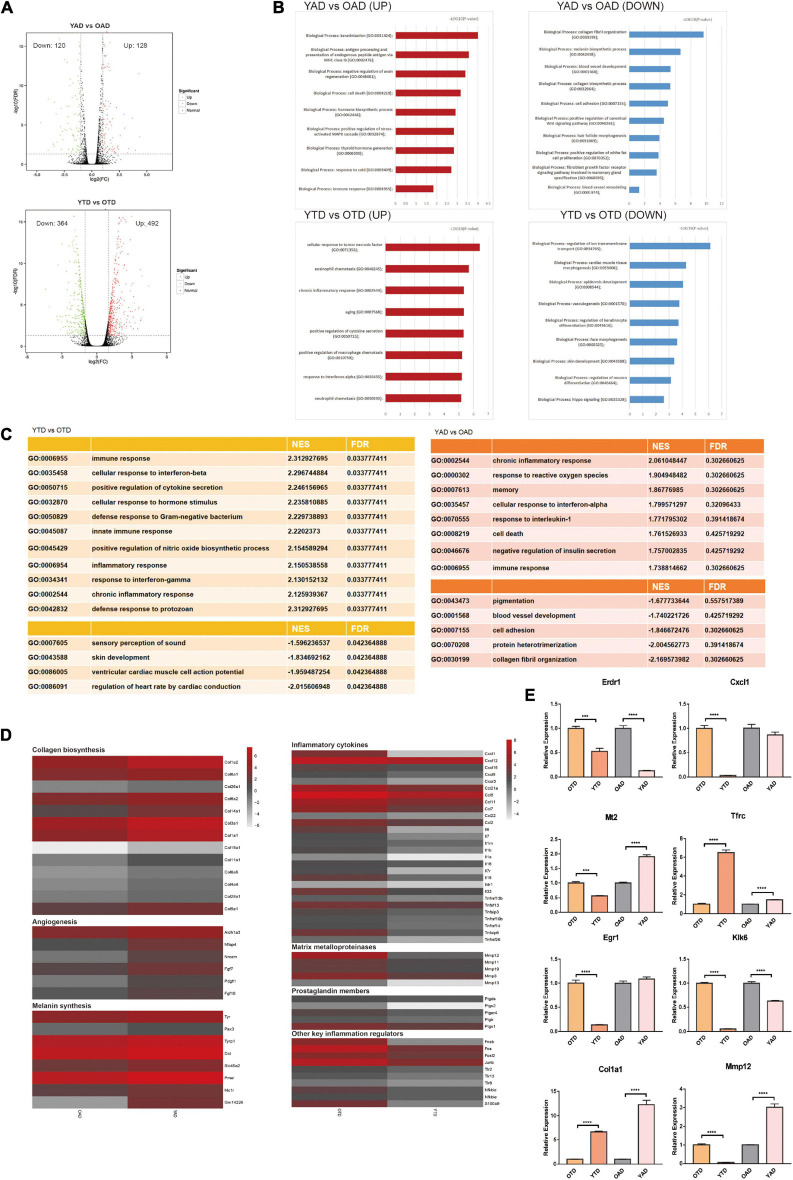
Overview of dWAT alterations with aging at anagen and telogen. **(A)** Volcano Plots of DEGs in YTD vs OTD and YAD vs OAD. **(B)** GO enrichment analysis respectively for upregulated and downregulated DEGs in YTD vs OTD and YAD vs OAD. **(C)** GSEA analysis indicated the most significantly enriched terms in YTD vs OTD and YAD vs OAD. However, in YAD vs OAD, all terms’ FDR exceeds 0.05, which only represents general trends. **(D)** Heat map of genes related to collagen biosynthesis, angiogenesis, and melanin synthesis with significant changes in YAD vs OAD, and inflammation-related genes with significant changes in YTD vs OTD. Genes expressed at low levels are in gray, and genes expressed at high levels are in red. **(E)** qRT-PCR validation of selected significant DEGs (Klk6 and Erdr1are significant UP_DEGs and Col1a1and Mt2 are significant DOWN_DEGs in YAD vs OAD; Cxcl1, Egr1, Mmp12 are significant UP_DEGs and Tfrc is significant DOWN_DEG in YTD vs OTD). For each experimental data point, *n* = 8. ***P < 0.001, and ****P < 0.0001.

After performing differential expression analysis for pairwise comparisons—YTD vs OTD and YAD vs OAD, we found that the number of differentially-expressed genes (DEGs) in the YTD vs OTD comparison is much higher than that in YAD vs OAD (856 vs 248, Figure 2A). In old mice, HFs are trapped in long-period telogen and seldom initiate anagen spontaneously. In a previous section, we found that aged HF still retained regenerative abilities for anagen induction upon stimuli, underscoring the main difference between aged and young HF was in telogen. In agreement with this, when comparing aged dWAT with young dWAT, more differences were identified in telogen than anagen.

To broadly determine the biological function changes of dWAT with HF cycling during aging, we performed GO enrichment analysis respectively for upregulated and downregulated DEGs of the above comparisons. Consequently, in anagen, transcripts for proteins involved with “collagen fibril organization,” “melanin biosynthetic process,” “blood vessel development,” and “cell adhesion” were the top functional categories of genes decreased in OAD (Figure 2B), which was consistent with the GSEA conclusions (Figure 2C). In anagen, the expansion of dWAT was accompanied by tissue reconstruction with collagen synthesis (Yamamoto and Yamauchi, 1999), vascularization (Yano et al., 2001), and so on. Nonetheless, aging dWAT exhibited important molecular features of impaired collagen biosynthesis, angiogenesis, and melanin synthesis while in anagen (Figure 2D), which indicated great structural and compositional remodeling and a reduced capacity for tissue regeneration in aging dWAT. On the one hand, these discoveries suggested the function degradation of cells in dWAT with aging, and on the other hand, ECM remodeling, in turn, disturbed the homeostasis of cells inside dWAT and neighboring skin cells, such as HF cells.

In telogen, the level of inflammation-related genes was massively increased in aged dWAT. The top upregulated biological progress GO terms in YTD vs OTD which were congruously enriched in inflammation activities were: “cellular response to tumor necrosis factor,” “eosinophil chemotaxis,” “chronic inflammatory response,” and “positive regulation of cytokine secretion” (Figure 2B). GESA results also proved significant enriched activated inflammation programs in OTD (Figure 2C). We therefore concluded that inflammatory infiltration was the most striking feature distinguishing OTD from YTD. Specifically, substantial inflammatory cytokines and genes related to matrix metalloproteinases, prostaglandin biosynthesis, positive immune regulation, and activator protein 1 (AP-1) family were all included in the most predominant upregulated DEGs in OTD (Figure 2D).

Besides, normal basic biological functions were also altered in aged dWAT at telogen. Downregulation of “regulation of ion transmembrane transport,” “epidermis development,” “regulation of keratinocyte differentiation,” and “skin development” supported the idea that reprogrammed aged dWAT failed to provide essential growth signals to neighboring skin cells (Figures 2B,C). Finally, to validate this RNA-Seq data, differential expression levels of some significant signature genes were further evaluated by RT-qPCR in samples obtained from independent biological replicates (Figure 2E).

### Aggravated Inflammation of Aging Dermal White Adipose Tissue During Telogen Perturb Tissue Homeostasis

**FIGURE 3:**
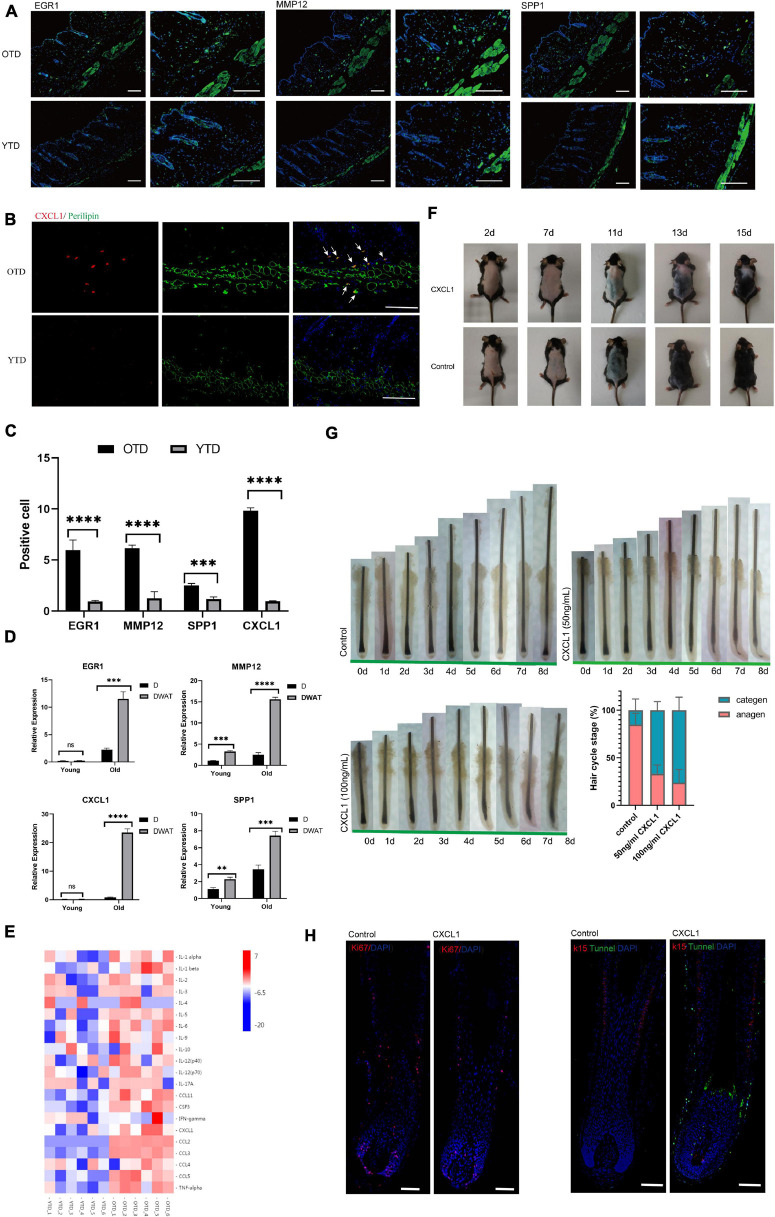
Aberrant inflammatory infiltration in aging dWAT during telogen. **(A–C)** Immunostaining images showed enhanced expression of crucial inflammation regulators EGR1, CXCL1, MMP12, SPP1 in OTD. Their distribution was mainly concentrated in dWAT. CXCL1 co-located with perilipin, a marker of mature adipocytes. Scale bars = 100 μm. **(D)** The relative mRNA expression level of the differential genes EGR1, CXCL1, MMP12, SPP1 in dermal tissues and dWAT. *n* = 8. **(E)** Significant upregulated protein levels of 21 inflammatory cytokines were found in OTD by a mouse cytokine/chemokine 23-plex array panel. *n* = 6. **(F)** CXCL1 injection resulted in delayed HF activation. **(G)** Organ culture assays of hair follicle. Human isolated hair follicles cultured with CXCL1 for 8 days in 24 well plates and histomorphometric analysis of hair follicle stages after organ cultured 4 days. Addition of CXCL1 50 and 100 ng/mL promoted hair follicle entry categen. For each experimental data point, *n* = 12. **(H)** Immunofluorescence of Day 3 cultured hair follicle showing apoptosis and decreased proliferation signaling appeared significantly in the CXCL1 group. K15, a specific marker of HFSC, was co-stained with apoptosis signal using tunnel test kit as well. Scale bars = 200 μm. **P < 0.01, ***P < 0.001, and ****P < 0.0001.

A continuous, low-grade inflammation typical of aging, which has been termed as “inflamm-aging,” perpetrates adverse tissue structural and functional remodeling in age-related diseases (Xia et al., 2016; Lu et al., 2021). Comparing with YTD, we found that substantial inflammatory cytokines [such as chemokines, interleukins, and tumor necrosis factor-alpha (TNFalpha)] significantly elevated during telogen in OTD. To further demonstrate the aberrant expression levels of inflammatory regulators in OTD, we selected some important inflammation-related signature DEGs, including CXCL1, MMP12, EGR1, and SPP1, which all were most significantly overexpressed in OTD, to perform immunofluorescence. Obviously, we obtained very similar results showing that these proteins in OTD assume overexpression significantly compared with that in YTD, and their distribution was mainly concentrated in dWAT (Figures 3A–C), and which was consistent with the results of qRT-PCR (Figure 3D). Furthermore, CXCL1 and MMP12 both indicated high intravascular expression in OTD. Hence, a significantly aggravated inflammation infiltration of aging dWAT during telogen was identified.

Next, using KEGG Pathway enrichment analysis, we found that the TNF signaling pathway was one of the most significant activated pathways in OTD (Supplementary Figure 2). In addition to TNF signaling, many chemokines and interleukins were upregulated markedly in OTD. The accumulation of these cytokines would exert chemotactic activities for macrophages (Cx3cl1, Ccl11, Ccl2), neutrophil (Il1b, Cxcl9, Ccl2, Cxcl1), and eosinophil (Ccl8, Ccl11, Ccl2, Ccl7), highlighting the locally advanced inflammation response in aging dWAT during telogen (Figure 2D). In obesity, adipocytes produced several chemokines (Cxcl2, Cxcl12, Ccl2, etc.) recruiting macrophage and neutrophils, causing systemic inflammation, insulin resistance, and the dysfunction of local endothelial cells (Gouranton et al., 2011; Rouault et al., 2013; Kim et al., 2014). Therefore we deduced that in aging dWAT, concentrated secretion of inflammatory cytokines in telogen would play a key role in HF disability. To verify the secretion of the above cytokines in OTD, we used a mouse cytokine/chemokine 23-plex array panel. As a result, significantly upregulated protein levels of 21 cytokines were found in OTD, including TNFa, CXCL1, CCL2, and interleukins (Figure 3E), highlighting the enhanced production of these inflammatory cytokines in OTD and their critical role in HF modulation.

Previous studies proved an age-associated increase of inflammatory cytokines derived from the epidermal compartment contributed to aging phenotypes of HFSCs (increased number, impaired function, and inability to tolerate stress) (Doles et al., 2012). Likewise, it was plausible that the inflammation signaling of aged dWAT during telogen would inevitably destabilize HFSCs maintenance. We have already verified the massively increased secretion of inflammatory cytokines in OTD, and here we further summarized specific functional mechanisms of these known inflammatory mediators during HF development in Table 1, which suggests various possible negative regulation mechanisms of inflammatory factors on HFs.

**TABLE 1:** Functional mechanisms of known inflammatory mediators in HF development and specific expression patterns in aging dWAT.

Cytokines	Properties related to HF	References	Expression patterns in dWAT_FPKM		
			Gene	OTD	YTD
TNF-a	Have significant positive association with AA; is required for normal cell death of HF and anagen-catagen transition in mice; Injection of TNF-a leads to apoptosis of HF bulb matrix keratinocytes in mice. TNF-a also inhibits HF elongation in a dose-dependent manner. K14-TNF alpha transgenic mice exerted impaired HF growth.	Cheng et al. 1992; Philpott et al. 1996b; Soma et al., 1998; Ruckert et al., 2000; Tong and Coulombe 2006; Perez-Garijo et al. 2013; Atwa et al. 2016	Tnfaip6 Tnfrsf13b Tnfaip8l2 Tnfsf13 Tnfaip3 Tnfrsf10b Tnfrsf11a Tnfrsf1b Tnfsf10 Tnfrsf14 Tnfsf12 Tnfrsf26	9.95549 1.87504 7.895 11.0502 3.83357 2.64808 2.01242 10.7332 9.14176 2.82864 39.4577 1.20813	1.71115 0.630668 5.12489 5.9425 1.64293 1.14413 1.15726 6.5328 5.90121 1.09449 29.5722 0.199513
IL-6	IL-6 KO mice resulted in STAT3 pathway activation and enhanced wound-induced hair neogenesis; DP cells secreted IL-6 in response to dihydrotestosterone.	Yu et al. 2008; Kwack et al. 2012; Nelson et al. 2016	Il6st Il6 Il6ra	111.495 6.19491 7.08773	72.9043 0.224645 5.37911
IL-1a/b	Act as a crucial mediator inducing HF regression. Upregulation of IL1 leads to diminished and atrophic hair follicles; IL-1a and IL-1b inhibit HF growth in organ culture; IL1 transgenic mice were characterized by hair loss and focal skin inflammation.	Harmon and Nevins 1993; Groves et al. 1995; Philpott et al. 1996a; Hoffmann et al. 1997; Hoffmann et al. 1998	Il1a Il1b Il1rap Il1r1 Il1r2 Il1rn Il1rl2	0.514973 3.36946 2.6094 11.1733 9.15598 2.50242 5.44209	0.0371823 0.739462 2.00408 7.76998 6.54273 1.32742 3.64348
IL16	Its polymorphisms may play a role in AA;	Lew et al. 2014	Il16	3.20473	1.97227
IL-7	IL-7 derived from HF are required for homeostasis of CD4(+) and CD8(+) skin-resident memory T cells	Adachi et al. 2015	Il7 Il7r	2.87597 2.72528	0.598882 0.162671
IFN-gamma	An important inducer of catagen in HF; its upregulation can result in the collapse of immune privilege of HF	Ito et al. 2005; Harries et al. 2013; Ito 2013; Ryu et al. 2014	Ifnar2 Ifngr1	36.3087 55.3079	23.0931 40.3651
Cxcr3	Overexpressed on alopecic effector T cells and its ablation prevents AA onset; Deficiency in cxcR3 impaired the patterning of primary hair placodes.	Lefebvre et al. 2012; Dai et al. 2016	Cxcr3	1.09397	0.289984
CXCL9/10/11	Significantly upregulated in AA lesions and promoted AA progression.	Suarez-Farinas et al. 2015; Dai et al. 2016	Cxcl9 Cxcl10 Cxcl11	2.91259 7.33465 0.0975968	1.33173 4.84593 0.0488607
Cxcl1/2	Cxcl1/2 are associated with AA susceptibility	Kim et al. 2015	Cxcl1	15.1405	0.108917
			Cxcl2	0.781783	0.158399
Ccl2	Plucking HFs released Ccl2 to signal to neighboring unplucked HFs, activating HFSCs for regeneration; the isthmus of HF expressed CCL2 to promote the recruitment of Langerhans cells into skin	Nagao et al. 2012; Chen et al., 2015	Ccl2	15.5977	5.95203
CCL8	HF bugle region produced CCL8 which inhibit the recruitment of Langerhans cells into the skin	Nagao et al. 2012	Ccl8	269.611	87.7888

Furthermore, we found that CXCL1 was one of the top significant upregulated DEG in OTD, and its overexpression in aging dWAT was further confirmed by immunofluorescence and cytokine array panel. CXCL1 co-localized with perilipin, a marker of mature adipocyte, in dWAT (Figure 3B). To investigate whether CXCL1 evokes alopecia, C57 mice received an intradermal injection (500 ng in 500 μl) every other day for a total of five times. Observably, the CXCL1 group indicated a delayed telogen-anagen transition (Figure 3F). In order to identify that CXCL1 had direct inhibitory effects on hair growth, we performed organ culture assays with isolated human hair follicles in the presence of CXCL1 (50ng/mL and 100ng/mL) or PBS. As a result, CXCL1 inhibited hair growth and promoted hair categen entry *in vitro* (Figures 3E,F). CXCL1 induced most hair follicles started to enter categen in the third day at 50 ng/mL or in the second day at 100 ng/mL, while most hair follicles in the control group were still in anagen status until the eighth day (Figure 3G). Using immunofluorescence, we also found that apoptosis in the CXCL1 group rose significantly in the hair matrix and connective tissue sheath, in addition, CXCL1 decreased the Ki67 signaling (Figure 3H). These results suggested that CXCL1 could promote cell apoptosis and prevent the cells’ proliferation of hair follicles. The inhibition function of CXCL1 on HFs underlined its negative role in the process of aging dWAT regulating HFs. Thus, we inferred that infiltrated inflammation factors of aging dWAT played an inhibitory role together, resulting in the hindrance of HF growth.

### Wnt, FGF, and BMP Signaling Altered in Aging Adipogenic Macroenvironment

**FIGURE 4:**
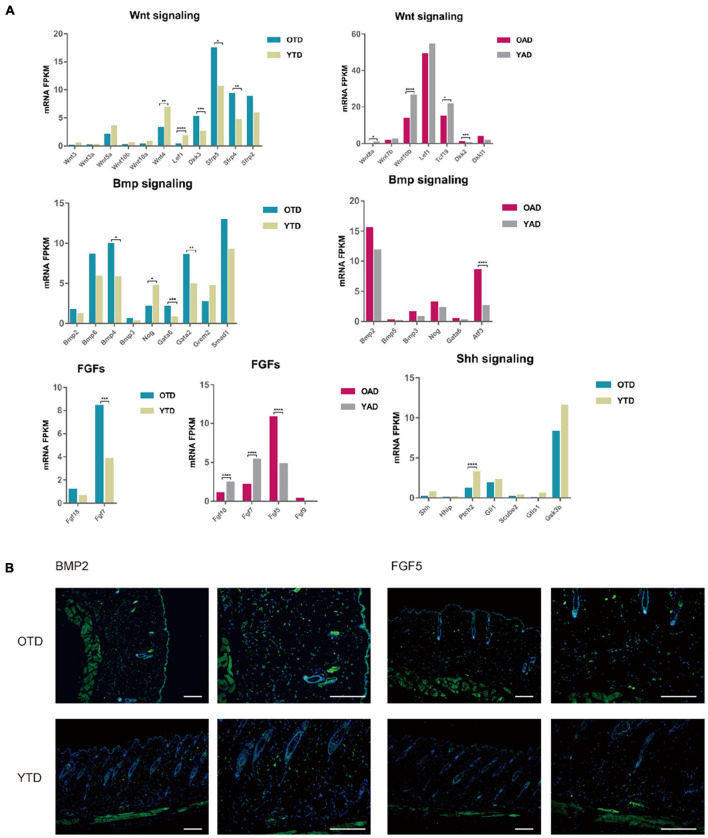
Aging dWAT negatively modulated HF behaviors. **(A)** Essential growth signaling for HF development indicated abnormal expressions in aging dWAT, including WNT, BMP, and FGFs. SHH-related signaling showed little changes. **(B)** BMP2 and FGF5 indicated overexpression in aging dWAT. Scale bars = 100 μm. *P < 0.05, **P < 0.01, ***P < 0.001, and ****P < 0.0001.

It is well known that dWAT can secrete some critical regulator molecules that participate in the regulation of HF homeostasis, including BMPs (Plikus et al., 2008), PDGF (Festa et al., 2011), DKK1 and Sfrp4 (Chen et al., 2014), and HGF (Nicu et al., 2021). Here, in addition to the identified inflamm-aging of dWAT, we wondered if other essential signalings supporting HF growth were also extensively reprogrammed. Therefore, we analyzed and summarized the expression level of the regulatory signal molecules of HF. Consequently, we found, Wnt signaling pathway, FGFs, as well as the BMP signaling pathway all indicated abnormal alterations in aging dWAT (Figure 4A).

Canonical WNT signaling was one of the most crucial regulators of dWAT and HF development. In the data, repressed WNT signaling in both anagen and telogen of aging dWAT was reported. Active WNT signaling was a vital step for HF to initiate new anagen (Greco et al., 2009). Comparing with YAD, obvious downregulation of WNT10b and Wnt8a, and upregulation of DKK2 indicated inhibited WNT signaling in OAD. In accordance with anagen, though with a lower expression level, WNT signaling of telogen also downregulates in OTD relative to YTD, including downregulation of WNT3, WNT3a, and LEF1, and upregulation of WNT inhibitors DKK3 and Sfrp4 (Figure 4A). Sfrp4 has been proven previously to be upregulated in aging dWAT, leading to HF regeneration retention (Chen et al., 2014). All of these results underscored an age-dependent repression of WNT signaling in dWAT, which might be partially responsible for the diminished self-renewing capacity of aging HF.

Furthermore, we noted a significant change in FGFs in aging dWAT, especially during anagen (Figure 4A). FGF5, an essential inhibitor of HF growth, which induced HF regression and promoted the transition from anagen to catagen (Hebert et al., 1994; Higgins et al., 2014), now indicated elevated expression in aging dWAT during anagen, which may contribute to the retarded HF growth. Additionally, downregulation of FGF7 and FGF10 was detected in OAD as well. As major HF regulators, FGF7 and FGF10 peaked at anagen V, playing a crucial role in stimulating HF development (Kawano et al., 2005; Iino et al., 2007). Taken together, the results presented showed that the aging dWAT secreted an increased level of inhibitory FGF signal, FGF5, and a decreased level of positive FGF signal, FGF10, and FGF7, which makes it difficult for aging HFs to activate and enter anagen.

Besides, it was well established that enhanced BMP2 expression of adipocytes resulted in a prolonged refractory telogen (Plikus et al., 2008). In our results, BMP2 also increased in both anagen and telogen, but not at significantly elevated levels (Figure 4A). Besides, BMP4, which was also released by dWAT and had an unfavorable effect on HF growth, indicated upregulation in OTD. Likewise, Noggin, a typical antagonist of BMP signaling, was also downregulated in OTD. Finally, we conducted immunofluorescence and proved the overexpression of BMP2 and FGF5 in aging dWAT, which may contribute to the impaired cycling behavior in aging mice (Figure 4B).

### Veratric Acid Facilitates Aging HF Regrowth by Mitigating Dermal White Adipose Tissue Inflammation

**FIGURE 5:**
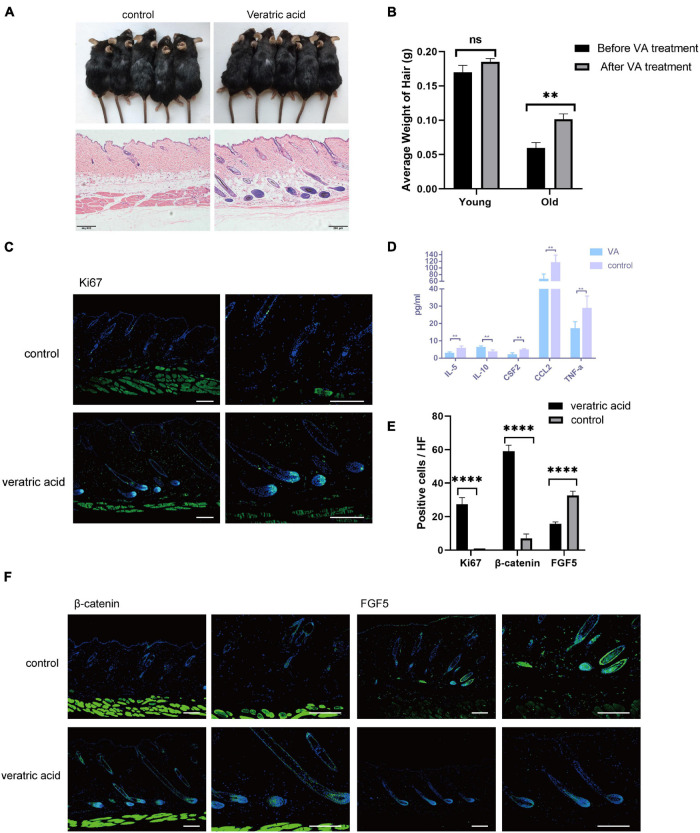
VA injection stimulated HF regrowth by lowering dWAT inflammation. **(A)** VA injection stimulated HF regenerative behavior in aged mice. **(B)** The weight of the back skin hair in the old mice after VA treatment was significantly heavier than that before VA treatment, while no difference was found in young mice. *n* = 10. **(C)** Increased ki67 signaling was identified in the VA group. Scale bars = 100 μm. **(D)** Decreased levels of TNF-a, CCL2, IL-5, CSF2, and increased IL-10 were proved in dWAT after VA treatment. **(E)** Quantification of positive cells in each hair follicle about ki67, β-catenin, and FGF5. **(F)** Increased β-catenin signaling and reduced FGF5 level were identified in the VA group. Scale bars = 100 μm. *n* = 6. ns, Not Significant. ^∗∗^*P* < 0.01, and ***P < 0.0001.

Collectively, based on the bioinformatics analysis, the current results unveiled aggravated inflammation and abnormal expression levels of HF regulatory signals of aging dWAT, all of which may play a negative role in the control of aging HF homeostasis. Here in the study, we focused on the dWAT inflammation. Then we ascertained whether the inflammation relief of aging dWAT would alleviate the aging HF retardation in telogen. We chose veratric acid (VA), a major benzoic acid derivative from plants and fruits, which has been proven to exhibit antibacterial, anti-inflammatory, and anti-oxidant activities (Choi et al., 2012; Shin et al., 2013; Ran et al., 2014), to perform intraperitoneal injection in aged mice. Finally, after 30 days, we noted that the gross appearance of hairs of the VA group became thicker, darker, and more lustrous than that of the control group (Figure 5A). Moreover, more anagen HFs in the VA group were identified based on histological H&E, corroborating activated HF growth (Figure 5A). Quantitative analysis showed that VA treatment increased the hair weight of old mice, and had no significant effect on young mice (Figure 5B). Meanwhile, increased Ki67 signaling was identified in the VA group (Figures 5C,E). Altogether, VA treatment can effectively facilitate aging HF regrowth. To further confirm whether VA reduced the inflammatory factor level of dWAT, the cytokine/chemokine 23-plex array panel was used for the test. The results further showed that the inflammatory cytokines were significantly reduced in the dWAT of the VA group, including TNF-a, CCL2, and IL-5. CSF2 and IL10 were increased (Figure 5D). However, the level of CXCL1 showed no obvious change (data not shown). There is some research confirming that VA could inhibit the generation of some inflammation cytokines, including interleukin-6 (IL-6), interleukin-8(IL-8), and interferon-γ (IFN-γ) (Choi et al., 2012; Shin et al., 2013; Wang Q. B. et al., 2016); so it is plausible that VA mainly downregulated the level of TNF-a, CCL2, and IL-5. CSF2 and upregulated the level of IL10 in aging dWAT, relieving the negative effect of these inflammatory factors on aging HFs and boosting HF regrowth.

We also tested the expression level of essential signalings for HF growth. Consequently, BMP signaling (BMP2/4) has no change after VA treatment (data not shown), but the enhanced level of β-catenin in HFs and decreased level of FGF5 of HFs and dWAT were proven (Figures 5E,F). Therefore, these findings suggested that the anti-inflammation effect of VA in dWAT was capable of finally attenuating the aging HFs dysfunction by activating β-catenin signaling and repressing the level of FGF5.

## Discussion

The local microenvironment plays a dominant role in determining cell behaviors. As an indispensable soil for HF growth, dWAT is closely bound up with HF homeostasis. Interpreting the interaction alterations between dWAT and HFs with aging is not only essential for understanding the involved aging mechanisms but also supply a promising therapeutic approach of senescent alopecia from the angle of dWAT targeting treatment.

**FIGURE 6:**
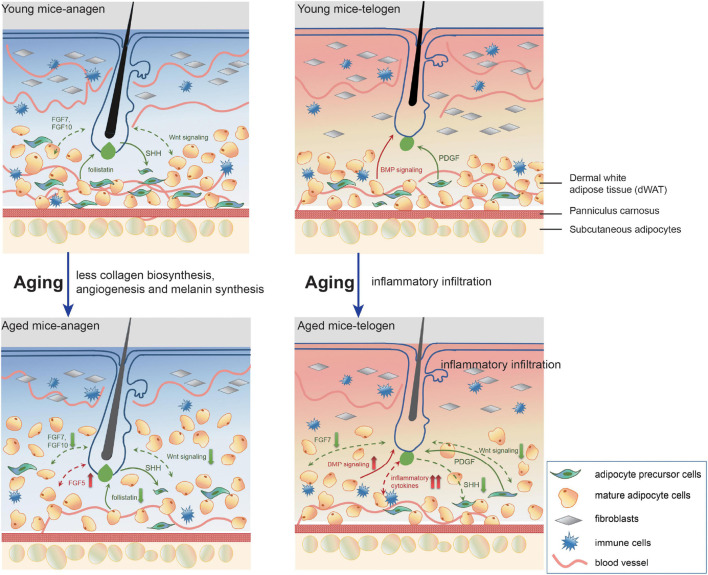
A model of pathological interrelationship alterations between dWAT and HF with aging. With aging, dWAT undergoes structural remodeling: the thickness increases with the replenishment of other transformed adipogenic lineage cells (aging dermal fibroblasts) (Salzer et al., 2018). Meanwhile, during anagen, the impaired ability of paracrine signals secretion of aging cells in dWAT resulted in deficient collagen biosynthesis, angiogenesis, and melanin synthesis. Besides, crucial regulators for HF development also indicated abnormal expression: including follistatin (Chen et al., 2014), WNT signalings, and FGFs. During telogen, remarkably, aberrant inflammation recapitulated aging dWAT, which caused a serious bottleneck for HF cycling. Concomitantly, altered BMP, WNT signalings, and FGFs also underscored the emerging role of dWAT in aging HFs malfunction. The green arrows represent positive effects and the red one represents negative effects. The solid and dashed lines denote published results and our results respectively.

Previous work has demonstrated that aged progenitor cells could be rejuvenated by exposure to a young systemic environment by establishing parabiotic pairings between young and aged mice (Conboy et al., 2005). In contrast, the regenerative potential of the progenitor cells of young animals was remarkably impaired after exposure to aging systemic milieu (Conboy et al., 2005). Furthermore, autologous skin graft transplantation experiments also proved that a young donor dWAT microenvironment could partially restore the regenerative cycling behavior of aged HFs (Plikus et al., 2008; Chen et al., 2014). In this study, we found that aging dWAT renovated tissue microenvironment, manifesting as inflammatory infiltration, ECM degrading, impaired collagen biosynthesis, angiogenesis, and melanin synthesis. This was accompanied by altered essential signalings (Wnt, Bmp) and growth factors (FGFs) for HF regulation. All these aging phenotypes reflect the function deterioration and tissue reconstruction of aging dWAT, and meanwhile, these altered factors are integrated to determine aging HF cell development (Figure 6).

In our profiling, plentiful collagen-related genes (Col1a1, Col3a1, Col6a6, Col5a1, Col4a4, etc.) occupied a large proportion of the decreased DEGs of OAD vs YAD. Collagen synthesis was mainly coupled to the anagen stage of the hair cycle (Plikus et al., 2008; Chen et al., 2014). Correspondingly, decreased expression of collagen-related genes in OAD indicated impaired the ability of collagen secretion with aging. Similarly, dermal vascularity increased with anagen and regressed during categen and telogen, which was orchestrated by HFs (Mecklenburg et al., 2000; Stenn and Paus, 2001). Perifollicular vascularization offered optimal growth nutrients facilitating HF growth and was positively related to HF size (Yano et al., 2001; Ozeki and Tabata, 2002). Deficiency of vascularization was also an important contributory factor of androgenetic alopecia (Chew et al., 2016). Impaired angiogenesis was tightly associated with the development of many age-related disorders (Ungvari et al., 2013; Roca et al., 2014) and also involved in OAD. Besides, decreased expression of key genes related to melanin synthesis in OAD was also identified, including Pmel, Mc1r, Slc45a2, and other melanocyte linage markers. This result might have two explanations: (1) adipose tissue had the function of melanin production and generated more melanin in obesity-related inflammation (Randhawa et al., 2009). The antioxidant and anti-inflammatory properties of melanin supported a hypothesis that melanogenesis plays an important role in abating oxidative stress and inflammation of adipose tissue (Randhawa et al., 2009). Here we speculated that aging weakened the melanogenesis of dWAT, causing increased oxidative stress and inflammation; (2) HF melanogenesis was strictly coupled to anagen, ceased during categen, and was absent in telogen (Slominski et al., 1994). Impaired melanogenesis here might also come from the unavoidable mixed hair bulbs in the isolated tissues and was in agreement with the extensively reduced melanin production of graying HFs. Collectively, all these phenomena could be attributed to the decrease in paracrine signals secretion of aging cells in dWAT. It is noticeable, however, there is not a one-way communication between HF and dWAT. Aging HFs might also have significant implications on the defective performance of aging dWAT, which is worth deeper exploration.

The most prominent pathological characteristic of aging dWAT was aberrant inflammatory infiltration in telogen. We have identified increased expression levels of substantial inflammatory cytokines, TNFa, CXCL1, CCL2, interleukins, as well as significant inflammation regulators, including MMP12, EGR1, and SPP1 in OTD. Moreover, we found that compared to the epidermis and dermis layer, these inflammation regulators are mainly concentrated in dWAT. It raised the tantalizing possibility that in aging dWAT, continuous production of inflammatory cytokines caught HF in prolonged telogen and suppressed HF cycling behavior. Among these inflammation regulators, heightened TNF production was an essential part of the inflammatory microenvironment of aging diseases (Slominski et al., 1994). The organ culture system has confirmed that TNFalpha markedly inhibited HF elongation in a concentration-dependent manner, resulting in morphological abnormalities and bulb matrix cells apoptosis (Soma et al., 1998; Ruckert et al., 2000). Here we focused on CXCL1, one of the top DEGs in OTD, which showed a significantly high intravascular expression in aging dWAT. Using an *in vivo* mouse model, we proved that intradermal injection of CXCL1 delayed HF cycling behavior. Meanwhile, organ culture with CXCL1 revealed similar results. In the data, we noted that the level of CXCL1 did not downregulate in anagen (Figure 2E). Nonetheless, its negative role was not significant at anagen. There may be two reasons. One is because the expression level of other activation signals exceeds the level of CXCL1, so the HFs are activated. Another reason may be because the anagen in our study was induced by depilation, which may be accompanied by upregulation of inflammatory factors activating HFs to enter anagen (Chen et al., 2015). Furthermore, the CXCL1 in OAD may be released by the plucked hair and may play an inductive role in anagen initiation rather than an inhibitory role. Overall, our data support a scenario in which overexpression of CXCL1 during telogen played a detrimental role in aging HF growth, trapping HF in telogen. Similarly, other inflammatory cytokines may also contribute to the aging HF retardation. We deduced that the synergistic effect of various inflammation regulators in dWAT triggered the dysfunctional growth of HFs.

Dermal white adipose tissue serves as an integral part of the skin and its stimulated inflammatory response in telogen with aging may result from the local microenvironment of the skin or systemic inflammation. In our study, we do not explore the detailed source of inflammation. From our perspective, the primary factor propelling basic aging processes was senescent cells in dWAT. Genes related to matrix metalloproteinases (MMPs), prostaglandin biosynthesis, positive immune regulators were included in the most predominant upregulated DEGs in OTD. With aging, senescent cells can secrete a complex combination of factors, including diverse cytokines, chemokines, growth factors, MMPs, and lipids, and this was referred to as the senescence-associated secretory phenotype (SASP) (Herranz and Gil, 2018; Di Micco et al., 2021), which is consistent with the phenotype of aging dWAT revealed by sequencing data. A new study has proven transplanting senescent adipose cells into young mice caused persistent physical dysfunction, including tissue inflammation (Xu et al., 2018). Our findings coincided with the possible contribution of SASP of the senescent cells to the inflamm-aging of dWAT. Therefore, like CXCL1, it can be inferred that the overexpression of these inflammation factors may be the consequence of SASP of dWAT. Meanwhile, the secretion of cytokines will recruit macrophages and may further secrete MMPs to degrade ECM. Besides, the degraded ECM also leads to the abnormal expression of inflammation factors. There are studies confirming that ECM can affect the secretion and expression of CXCL1 (Wang et al., 2018). So in conclusion, it can be inferred that both the overexpression of inflammation factors and ECM remodeling in aging dWAT are the result of changes in the microenvironment caused by aging, and the interplay of them further reinforced each other. Therefore, senescent cells, which are the source of SASP, are the most important targets. Recently, emerging studies supported selective elimination of senescent cells as a crucial therapeutic method for treating degenerative aging disorders (Baar et al., 2017; Childs et al., 2017; Jeon et al., 2017; Xu et al., 2018). These conclusions also indicated promising therapeutic approaches to ameliorate aging phenotype in dWAT.

With aging, a previous study proved that adipocyte cells overexpressed Bmp2, Dkk1, and Sfrp4, resulting in defective HF regeneration (Chen et al., 2014). Likewise, our results also identified aberrant expression of Bmp signaling and repressed Wnt signaling in aging dWAT in both anagen and telogen. Besides, some vital growth factors (FGFs) for HFs also downregulated in aging dWAT, highlighting the important contribution of the paracrine factors of dWAT to the diminished self-renewal of aging HFs. These aberrant signalings could serve as targets for the regulation of HF aging. However, since that the role of aggravated inflammation of aging dWAT on aging HFs has not been identified, we try to trigger HFs activation by temporarily lessening the inflammation activity. After high-level VA injection in aged mice, defective HF growth in aged mice was partially rescued, along with a significant downregulation of TNF-a, CCL2, IL-5, CSF2, and upregulation of IL10 in dWAT, further underscoring that targeting dWAT inflammation could activate aging HF cycling behavior. Moreover, VA treatment also activated β-catenin signaling and downregulated FGF5 expression level, but had no effect on BMP signaling. Altogether, VA injection alleviated the inflammation level of aging dWAT inflammation, interacting with other downstream signaling for HF growth, and finally induced aging HF regrowth.

Our study provides a detailed and direct portrait of dWAT alterations with aging and identifies its potential regulatory effect on aging HF. The most dominant feature of aging dWAT distinguishing young dWAT was massive inflammatory infiltration during telogen, which inevitably brought out deleterious influence and might be the core factor retarding HF regeneration. Moreover, reducing dWAT inflammation was capable to encourage HF cycling in aged mice. Nowadays, the crucial role of dWAT in HF regulation has been attracting more and more attention. Based on our study, future mechanistic work is needed for delineating the exact interplay between dWAT and HF with aging, which may provide new avenues for developing therapeutic agents to stimulate aging HF growth.

## Materials and Methods

### Ethics Approval and Consent to Participate

All experiments reviewed and approved by The Ethics Committee of Nanfang Hospital, Southern Medical University.

### Mice

To perform the aging study, young (2 months at telogen), and aged mice (16–18 months at telogen) C57BL/6 were utilized in this study. Mice were all provided by the Experimental Animal Center at Southern Medical University (Guangzhou, China). The experimental procedures were performed with protocols approved by the Institutional Animal Care and Use Committee.

### Sample Preparation for Transcriptome Sequencing

Using microdissection to separate dWAT tissue from skin. DWAT harvest from 20 young mice (2 months) and 20 aged mice (18 months) at telogen and 20 young mice (2 months) and 20 aged mice (18 months) at depilation-induced anagen were used for the transcriptome sequencing. Total RNA was isolated using Trizol reagent (Invitrogen, Carlsbad, CA, United States) and the RNA quality and quantity were evaluated by using Bioanalyzer 2100 and RNA 6000 Nano LabChip Kit (Agilent, CA, United States) with RIN number > 7.5. Qualified RNAs were used for further transcriptome sequencing analysis.

### RNA Library Construction and Sequencing

The library preparations were sequenced on an Illumina Hiseq 2500 platform and the details of library construction and sequencing are the same as in our previous study (Miao et al., 2018).

### Hair Follicle Organ Culture

Hair follicles in the anagen VI stage from normal human scalp skin in 12 persons were isolated as previously described (Philpott, 1999). Then the hair follicles were cultured for 8 days in Williams E medium supplemented with 2 mmol/L L-glutamine, 10 ng/mL hydrocortisone, 10 lg/mL insulin, and an antibiotic/antimycotic mixture at 37°C in 5% CO2 and 95% air in a humidified incubator as described previously (Philpott et al., 1994). To explore the role of CXCL1 in hair growth, the media were supplemented with recombinant human CXCL1 (Proteintech) at day 0. The normal medium was used as a control.

### Quantitative PCR Analysis

For qPCR validation of DEGs, we used another eight young mice (2 months) and eight aged mice (18 months) at telogen and depilation-induced anagen. RNA was prepared using the cDNA Reverse Transcription Kit (Promega, A5001, Madison, WI, United States). Aliquots of RNA were analyzed in triplicate by using qRT-PCR performed according to standard protocols by ABI Step One Plus system (Applied Biosystems, Foster, CA, United States) by GoTaq^®^ qPCR Master Mix (Promega, A6001, Madison, WI, United States). The melting curve was performed to verify the specificity of PCR amplification. RNA was normalized to expression levels of GAPDH. All data were analyzed by unpaired Student *t* test and were presented as the mean ± standard error. The Primer Sequences of qRT-PCR are as follow:

**Table:** 

Mus-GAPDH-F	ACCCCCAATG TGTCCGTCGT	Mus-Klk6-QF	AATGTAGTGACAC AGAGCACAGAAC
Mus-GAPDH-R	AGCCCAAGATG CCCTTCAGTGG	Mus-Klk6-QR	AACCACACCCA TAGAGTAGGATTG
Mus-Cxcl1-QF	CAAGTAACGGA GAAAGAAGACAGAC	Mus-Mmp12-QF	CAAAGTCAATAA TGTACCCCACC
Mus-Cxcl1-QR	AGGACCCTCAAA AGAAATTGTATAG	Mus-Mmp12-QR	CCATAGAGGGA CTGAATGTTACG
Mus-Col1a1-QF	TCTCTGGTCT CCAGGGTCCTC	Mus-mt2-QF	GGGCTGCATCT GCAAAGAG
Mus-Col1a1-QR	TCCGTCTTTG CCAGGAGAAC	Mus-mt2-QR	AAAGGCTAGGC TTCTACATGGTC
Mus-Egr1-QF	CTCCTTCAGC ACCTCAACTGG	Mus-Tfrc-QF	AATCAAAATGTGA AGCTCATTGTG
Mus-Egr1-QR	CTTCCCTCCTGT GCTTTTATGTC	Mus-Tfrc-QR	TGGGCTCCTACTA CAACATAACG
Mus-Erdr1-QF	CGAAAGCACA CACGTAGAAGC	Mus-Spp1-QF	TCACTCCAATC GTCCCTACA
Mus-Erdr1-QR	CTTCCTCCGTG AGAATCGCT	Mus- Spp1-QR	CTGGA ACATCG TATGGGTA

### Immunofluorescent Staining and Confocal Microscopy

The samples were fixed in 4% paraformaldehyde, dehydrated, then embedded in paraffin and sectioned (4–6 μm).

All procedures were performed according to the standard protocol. Antibodies and dilutions used were: CXCL1 (1:100, proteintech), MMP12 (1:50, proteintech), SPP1 (1:50, Abcam), EGR1 (1:50, Abcam). β-catenin (1:250,Abcam), BMP2 (1:100, Abcam), FGF5 (1:100, Abcam), ki67 (1:250,Abcam), Secondary antibodies: Alexa Fluor-488 (Abcam). Nuclei were stained using 4 diamidino-2-phenylindole (DAPI). Proliferation and apoptosis of hair follicles were assessed by Ki67/TUNEL immunostaining as reported before (Foitzik et al., 2006). Imaging was performed using Zeiss LSM 880 confocal microscope (Zeiss, Oberkochen, Germany).

### CXCL1/Veratric Acid Treatment

CXCL1 (500 ng) was dissolved in 500ul 0.9% NaCl and intradermally injected in the dorsum of 6–7 week C57 mice (*n* = 10 per group). The control group (*n* = 10 per group) were injected with 500 μl solvent. The injection treatment was conducted every other day for a total of five times.

Veratric acid (80 mg/kg) dissolved in 2% DMSO + 40% PEG + 5% Tween + H_2_O, and were injected in 26 aged C57 mice (16 months) once per day for 30 days, the control group are 20 of the same aged mice (16 months), which intraperitoneally received an equal volume of 2% DMSO + 40% PEG + 5% Tween + H_2_O per day for 30 days as well. The mice were killed for further experimentation at 30d.

### Luminex Cytokine Panels

A mouse Bio-Plex cytokine/chemokine 23-plex array panel based on Luminex technology was used for the quantification of inflammatory cytokines/chemokines in dWAT. DWAT harvest from six young mice (2 months) and six aged mice at telogen were analyzed in the panel. Besides, dWAT in six mice from the VA group and six mice from the control group were also analyzed in the panel. Data were measured on Bio-Plex 200 System and calculated by Bio-Plex Manager 6.0 and 6.1 software.

## Data Availability Statement

The datasets presented in this study can be found in online repositories. The names of the repository/repositories and accession number(s) can be found below: https://www.ncbi.nlm.nih.gov/, GSE134893.

## Ethics Statement

The studies involving human participants were reviewed and approved by The Ethics Committee of Nanfang Hospital, Southern Medical University. The patients/participants provided their written informed consent to participate in this study. The animal study was reviewed and approved by The Ethics Committee of Nanfang Hospital, Southern Medical University.

## Author Contributions

JC: design, methodology, writing, data collection, and analysis. Z-XF and D-CZ: methodology, data collection, and design. Y-LG and KY: methodology and formal analysis. DD and ZG: data curation and formal analysis. Z-QH: study design and methodology. YM: funding acquisition, supervision, and writing—review and editing. QQ: project administration, funding acquisition, design, supervision, data collection and analysis, and writing—review and editing. All authors agreed with the manuscript content.

## Conflict of Interest

The authors declare that the research was conducted in the absence of any commercial or financial relationships that could be construed as a potential conflict of interest.

## Publisher’s Note

All claims expressed in this article are solely those of the authors and do not necessarily represent those of their affiliated organizations, or those of the publisher, the editors and the reviewers. Any product that may be evaluated in this article, or claim that may be made by its manufacturer, is not guaranteed or endorsed by the publisher.

## Supplementary Material

Supplementary Figure 1 Unsupervised clustering of OAD/YAD, OTD/YTD samples based on other adipose-related GO signatures.

Supplementary Figure 2 KEGG enrichment analysis respectively for upregulated and downregulated DEGs in YTD vs OTD and YAD vs OAD.
