# Supplementary figures and images for: Emerging Role of Dermal White Adipose Tissue in Modulating Hair Follicle Development During Aging
Source: Front Cell Dev Biol. 2021 Oct 15;9:728188. doi: 10.3389/fcell.2021.728188 (PMC8554130; doi:10.3389/fcell.2021.728188)

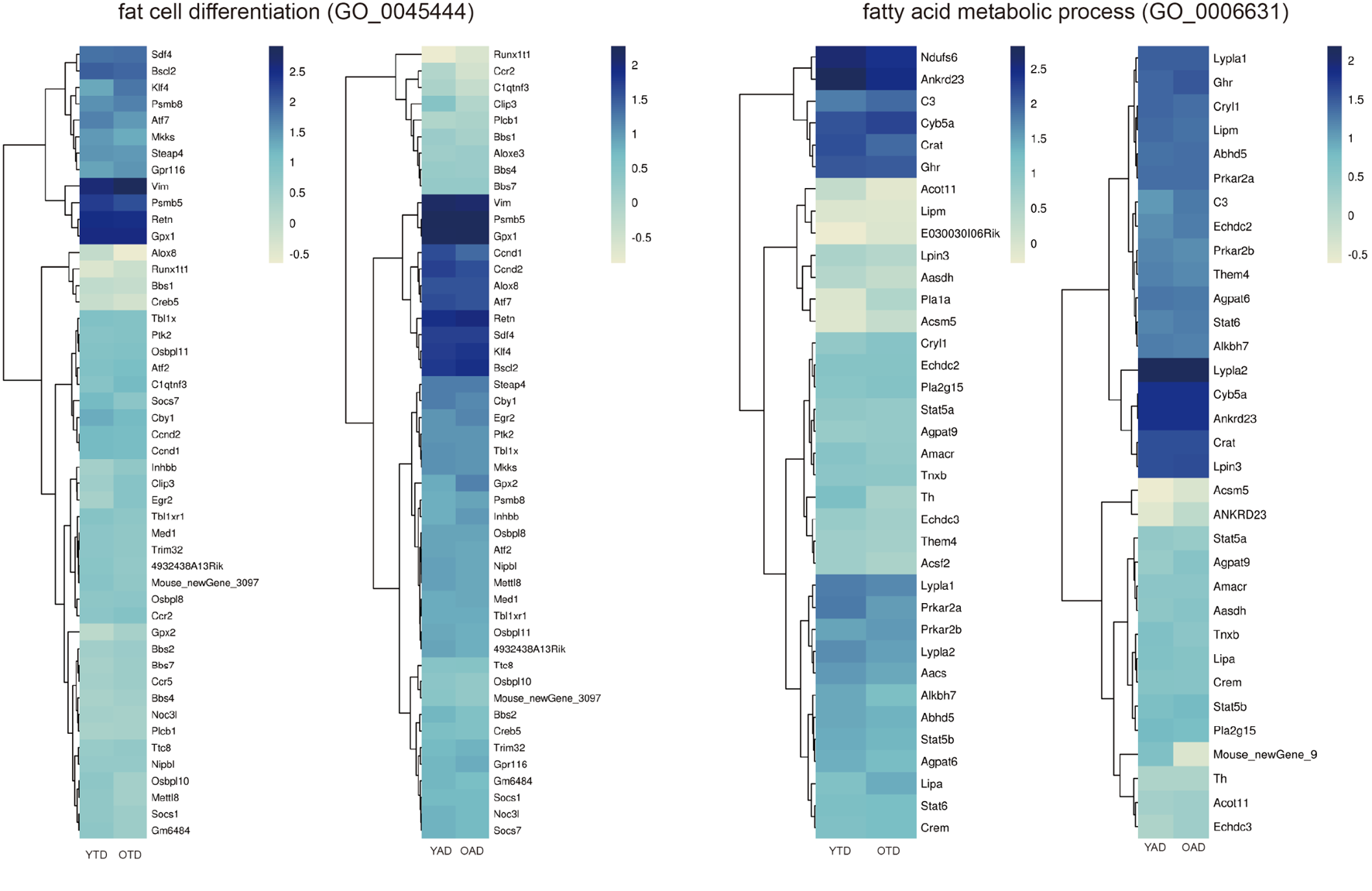

Supplement: Supplementary Figure 1 — Unsupervised clustering of OAD/YAD, OTD/YTD samples based on other adipose-related GO signatures. [file Image_1.TIF]

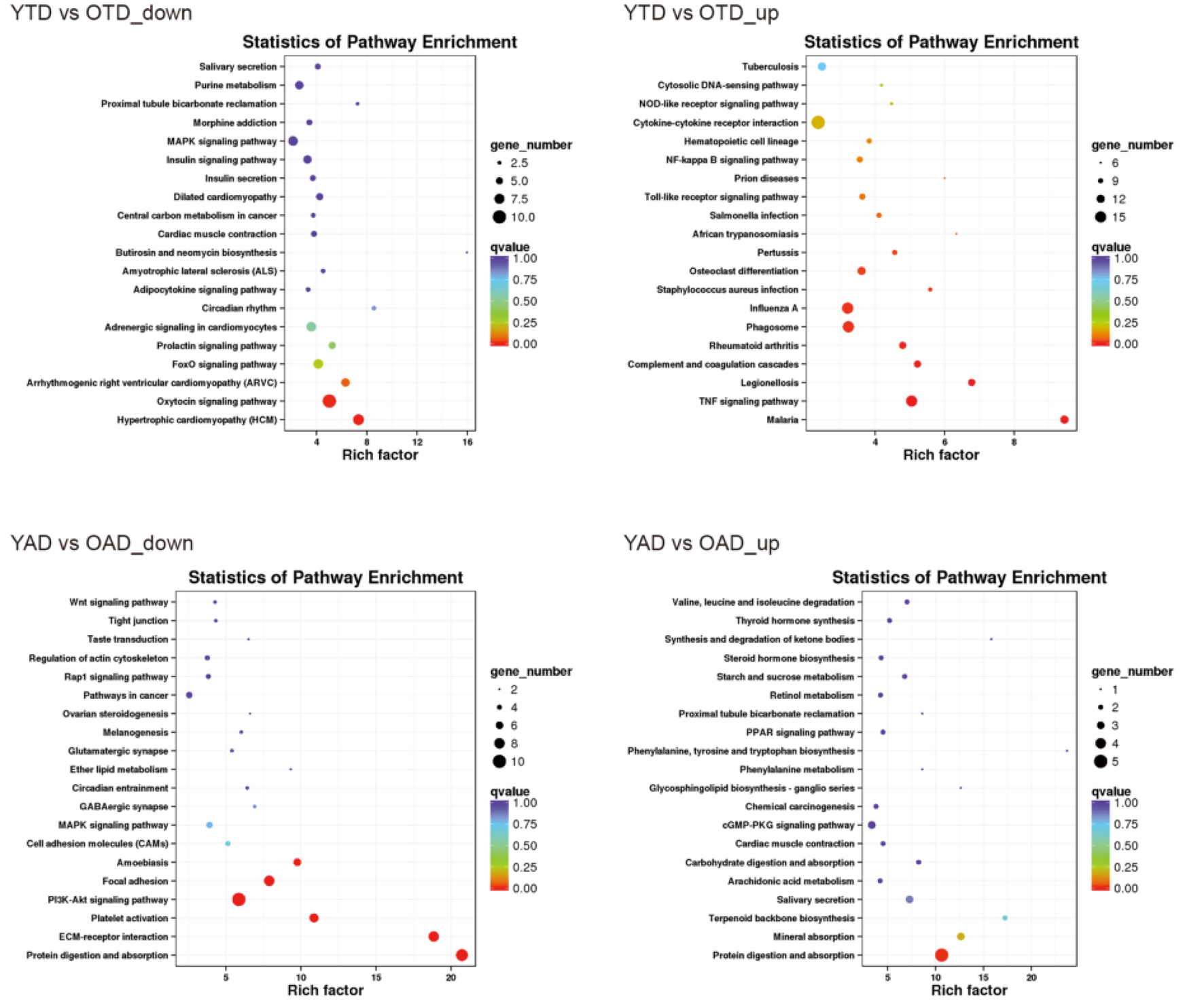

Supplement: Supplementary Figure 2 — KEGG enrichment analysis respectively for upregulated and downregulated DEGs in YTD vs OTD and YAD vs OAD. [file Image_2.TIF]
